# Modified high-flow nasal cannula oxygen therapy versus conventional oxygen therapy in patients undergoing bronchoscopy: a randomized clinical trial

**DOI:** 10.1186/s12890-021-01744-8

**Published:** 2021-11-14

**Authors:** Rui Wang, Hai-Chao Li, Xu-Yan Li, Xiao Tang, Hui-Wen Chu, Xue Yuan, Zhao-Hui Tong, Bing Sun

**Affiliations:** Department of Respiratory and Critical Care Medicine, Beijing Chao-Yang Hospital, Beijing Institute of Respiratory Medicine, Beijing Key Laboratory of Respiratory and Pulmonary Circulation Disorders, Beijing Engineering Research Centre for Diagnosis and Treatment of Respiratory and Critical Care Medicine (Beijing Chao-Yang Hospital), Capital Medical University, No. 8 Gongtinan Road, Chaoyang, Beijing, 100020 China

**Keywords:** Modified high-flow nasal cannula oxygen therapy, Conventional oxygen therapy, Bronchoscopy, Hypoxemia

## Abstract

**Background:**

Hypoxemia frequently occurs during bronchoscopy. High-flow nasal cannula (HFNC) oxygen therapy may be a feasible alternative to prevent the deterioration of gas exchange during bronchoscopy. With the convenience of clinical use in mind, we modified an HFNC using a single cannula. This clinical trial was designed to test the hypothesis that a modified HFNC would decrease the proportion of patients with a single moment of peripheral arterial oxygen saturation (SpO_2_) < 90% during bronchoscopy.

**Methods:**

In this single-center, prospective randomized controlled trial, hospitalized patients in the respiratory department in need of diagnostic bronchoscopy were randomly assigned to a modified HFNC oxygen therapy group or a conventional oxygen therapy (COT) group. The primary outcome was the proportion of patients with a single moment of SpO_2_ < 90% during bronchoscopy.

**Results:**

Eight hundred and twelve patients were randomized to the modified HFNC (n = 406) or COT (n = 406) group. Twenty-four patients were unable to cooperate or comply with bronchoscopy. Thus, 788 patients were included in the analysis. The proportion of patients with a single moment of SpO_2_ < 90% during bronchoscopy in the modified HFNC group was significantly lower than that in the COT group (12.5% vs. 28.8%,* p* < 0.001). There were no significant differences in the fraction of inspired oxygen between the two groups. The lowest SpO_2_ during bronchoscopy and 5 min after bronchoscopy in the modified HFNC group was significantly higher than that in the COT group. Multivariate analysis showed that a baseline forced vital capacity (FVC) < 2.7 L (OR, 0.276; 95% CI, 0.083–0.919, *p* = 0.036) and a volume of fluid instilled > 60 ml (OR, 1.034; 95% CI, 1.002–1.067, *p* = 0.036) were independent risk factors for hypoxemia during bronchoscopy in the modified HFNC group.

**Conclusions:**

A modified HFNC could decrease the proportion of patients with a single moment of SpO_2_ < 90% during bronchoscopy. A lower baseline FVC and large-volume bronchoalveolar lavage may predict desaturation during bronchoscopy when using a modified HFNC.

*Trial registration* ClinicalTrials. Gov: NCT02606188. Registered 17 November 2015.

**Supplementary Information:**

The online version contains supplementary material available at 10.1186/s12890-021-01744-8.

## Bacjground

Bronchoscopy has an important role in diagnosing and treating respiratory diseases and is now a useful tool to investigate abnormal pulmonary lesions [1]. Hypoxemia frequently occurs during bronchoscopy [2]. The partial pressure of arterial oxygen (PaO_2_) usually decreases by 10–20 mmHg during bronchoscopy [3], and bronchoalveolar lavage (BAL) is associated with an even greater decrease [4]. A previous report showed that oxygen therapy is required in 24% of patients during bronchoscopy [5].

To avoid bronchoscopy-induced hypoxemia, patients generally require oxygen therapy via a nasal cannula to maintain an arterial oxygen saturation (SaO_2_) > 90% during bronchoscopy [6]. Nasal cannulas supply oxygen based on the patient’s respiratory pattern, which limits their use. Compared with conventional oxygen supplementation, noninvasive ventilation (NIV) is known to prevent gas exchange deterioration during bronchoscopy in hypoxemic patients [7, 8]. However, NIV is seldom used because it is associated with ace mask intolerance, the difficulty of introducing the bronchoscope to the nares due to the face mask, and further complicated by the occurrence of patient-ventilator asynchrony, which may increase patients’ discomfort and intolerance.

High-flow nasal cannula (HFNC) oxygen therapy provides accurate oxygen delivery, wash-out of the anatomic dead space, and a low level of positive pressure [9]. HFNC is easy to use and well tolerated. Thus, HFNC therapy could be used as a new choice for oxygen therapy during bronchoscopy. Recently, HFNC has been shown to improve oxygenation in acute respiratory failure patients undergoing bronchoscopy [10–13]. In our center, the bronchoscope was passed through the nose during all procedures. HFNC oxygen therapy is applied to both nostrils. The bronchoscope occupies one of the nares receiving oxygen therapy during bronchoscopy. As a result, the application of HFNC needs to be optimized. We therefore designed a modified HFNC that has a single cannula.

We hypothesized that, during bronchoscopy, modified HFNC oxygen therapy may maintain oxygenation better than conventional oxygen therapy (COT). Therefore, we conducted a prospective randomized controlled study to determine whether a modified HFNC could decrease the proportion of patients with a single moment of peripheral arterial oxygen saturation (SpO_2_) < 90% during bronchoscopy.

## Methods

### Study design and patients

This was a single-center prospective randomized controlled trial (ClinicalTrials.gov, NCT02606188). Patients were recruited from the general wards of the Respiratory Department at Beijing Chao-Yang Hospital. This study was approved by the Ethics Committee of the Affiliated Beijing Chao-Yang Hospital, Capital Medical University (2015-KE-85), and informed consent was obtained from all patients or their legal guardians in writing.

We included patients who met the following criteria: (1) age more than 18 years old; and (2) indication for diagnostic bronchoscopy. Patients with any of the following criteria were excluded: (1) SpO_2_ < 90% on room air; (2) platelet count < 60 × 10^9^/L; and (3) nasopharyngeal obstruction or blockage.

### Randomization

At the time of admission, eligible patients were randomly assigned to the modified HFNC oxygen therapy group or the conventional oxygen therapy (COT) group for respiratory support during bronchoscopy. A randomization list in blocks of four was generated from a computer, and the treatment allocation was concealed using sequentially numbered, opaque, sealed envelopes. All nurses and other research personnel were blinded to the randomization schedule and block size.

Data collectors were aware of the study group assignments, but the analyses were performed by a research statistician who did not participate in the investigation and did not know the research groupings. Because of the nature of the intervention, physicians and nurses could not be blinded to the group assignments.

### Interventions

In the modified HFNC group, high-flow devices (AIRVO™ 2; Fisher & Paykel Healthcare, Auckland, New Zealand) were utilized for respiratory support. The nasal cannula used was a modified single nasal cannula. The modified HFNC was shown to have similar respiratory support characteristics as the regular HFNC in in vitro experiments (Additional file 1). The size of the nasal cannula was chosen based on the patient’s nostrils. The fraction of inspired oxygen (FiO_2_) was adjusted to maintain an SpO_2_ > 90%. The humidifier temperature was set to 37 °C, and the oxygen flow was set to 50 L/min.

In the COT group, oxygen was delivered via nasal prongs during bronchoscopy. The oxygen flow was set to achieve an SpO_2_ > 90%. COT provides 24%-45% oxygen with flow rates up to 6 L/min, although the patient’s respiratory pattern can influence the actual FiO_2_ [14]. We used a simple formula to estimate the FiO_2_, as follows: each 1 L/min of nasal O_2_ increased the FiO_2_ by approximately 4%.

In both groups, caregivers adjusted the FiO_2_ before hypoxemia occurred during bronchoscopy. The trigger to adjust the FiO_2_ was an SpO_2_ drop to 93%, and there was a downward trend. After bronchoscopy, patients continued to be given COT when SpO_2_ was < 90%.

### Flexible bronchoscopy and BAL

All bronchoscopy procedures were performed by three respiratory specialists, each with > 10 years of experience. Electrocardiogram (ECG), heart rate, respiratory rate, and SpO_2_ were recorded continuously by a bedside ECG monitor. The data collector recorded the vital signs on the paper case report form (CRF) after determining their authenticity. The data collector judged whether the vital signs were accurate based on their waveform and the patient's condition (e.g., agitation). If the data collector considered the vital signs to have artifacts, the patient was allowed to stabilize for a certain amount of time before re-recording. Blood pressure was monitored automatically and noninvasively every 5 min. Hypoxemia events were defined as SpO_2_ < 90% at a single time point. The worst values during bronchoscopy meant the lowest SpO_2_, highest FiO_2_, highest respiratory rate, highest heart rate, and highest mean arterial pressure.

**Fig. 1:**
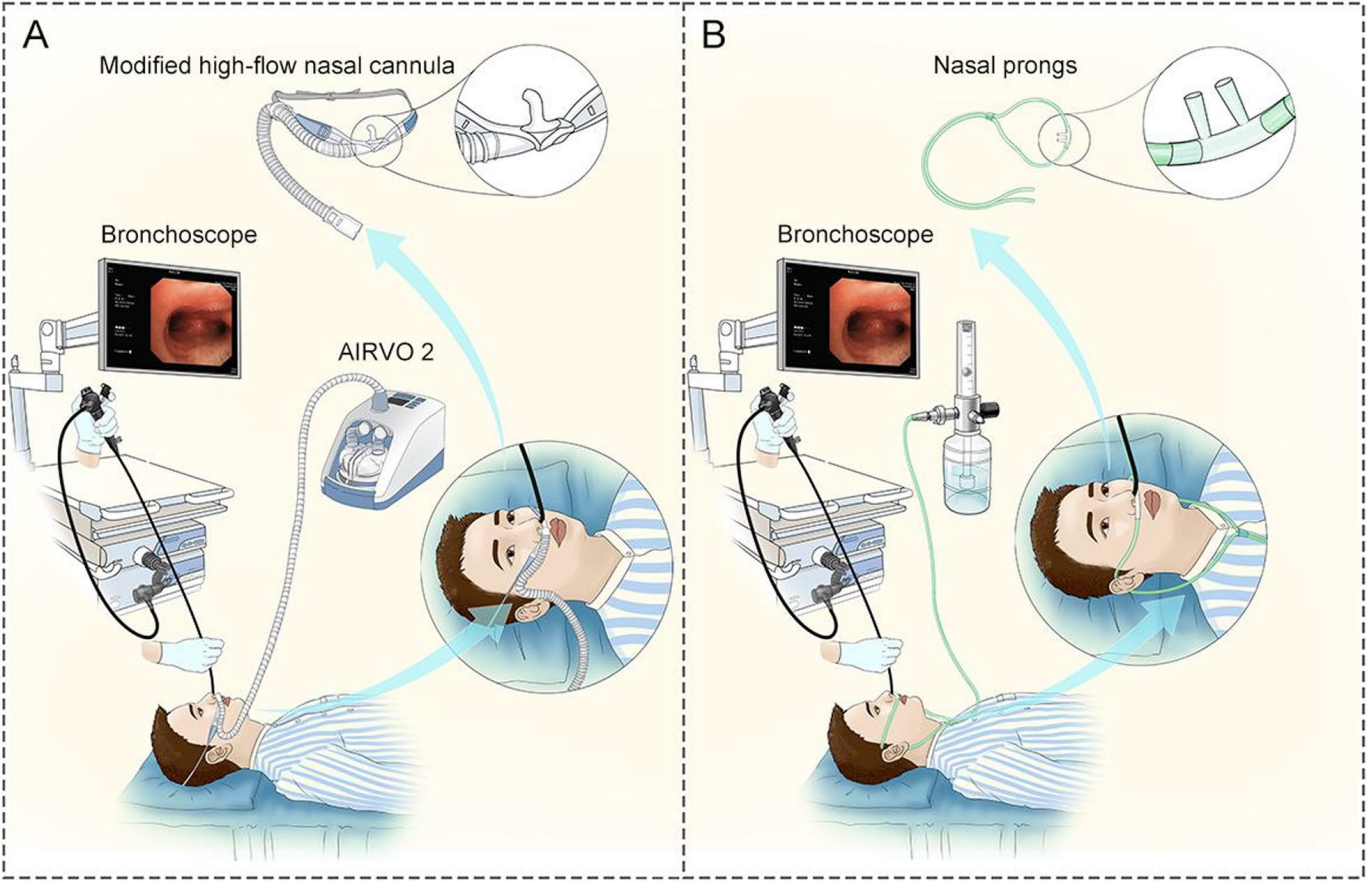
Illustration of bronchoscopy using a modified HFNC (**A**) or COT (**B**). *HFNC* high-flow nasal cannula, *COT* conventional oxygen therapy

The setup using modified HFNC oxygen therapy or COT is illustrated in Fig. 1. Patients in our study were given topical anesthesia, but no sedative was used. For topical anesthesia, 2% lidocaine was nebulized into the nasal cavity and pharyngeal mucosa. For all patients, a resting period of approximately 5 min was required for the local anesthesia to fully take effect. Bronchoscopy was performed transnasally with the patient in the supine position. The bronchoscope was inserted in the trachea, and then 10 ml of lidocaine was sprayed into the left and right main bronchi in aliquots of 5 ml. The bronchi were examined, and the bronchoscope was wedged into the appropriate segmental bronchus. BAL was performed using normal saline instilled in aliquots of 20 mL and then aspirated. The volume of BAL instilled depended on the category of disease and the patient’s condition. BAL fluid was sent for cytologic or microbiologic analysis.

The events during bronchoscopy included agitation, bronchospasm, arrhythmias, hypertension, epistaxis, and mucosal bleeding. Four main groups of arrhythmias, namely, extra beats, supraventricular tachycardias, ventricular arrhythmias, and bradyarrhythmia, were recorded. Hypertension was defined as systolic blood pressure > 180 mmHg.

The complications of bronchoscopy include fever, pneumothorax, and hemorrhage. A fever is defined as a temperature > 38 °C within the first 24 h after bronchoscopy [15]. Transient fever, which spontaneously resolves within 24 h, is the most common adverse event after BAL is performed. After a transbronchial lung biopsy was performed, a chest radiograph was routinely performed to determine whether the patient had a pneumothorax. In the case of significant pneumothorax, a chest tube was immediately inserted to avoid oxygen desaturation or tension physiology. Hemorrhage requires topical instillation of small amounts of adrenaline solution or more advanced interventions.

If the SpO_2_ persisted at < 90%, the examination was aborted or discontinued at the discretion of the bronchoscopist. Restart the procedure after the SpO_2_ has been restored to 90%.

### Endpoints and measurements

The primary endpoint was the proportion of patients with a single moment of SpO_2_ < 90% during bronchoscopy. The secondary endpoint was the duration of bronchoscopy, which was defined as the time between insertion and removal of the bronchoscope from the tracheobronchial tree [10]. Other endpoints were duration of SpO_2_ < 90% and the proportion of patients with procedural discontinuation. Other variables included the following: (1) demographic variables; (2) vital signs and FiO_2_ before bronchoscopy, the worst values during the procedure and within 24 h after bronchoscopy; and (3) bronchoscopy-related events and complications.

### Statistical analysis

#### Sample size estimation

A sample size of 390 participants per group was chosen to have 80% power to demonstrate that the modified HFNC group was superior to the COT group for the primary measure (proportion of patients with an SpO_2_ < 90% during bronchoscopy), with the use of a margin of 0.08 based on an observed 28% of patients with an SpO_2_ < 90% during bronchoscopy for the COT group in a previous study and an assumed 20% for the modified HFNC group [16].

#### Comparisons of the two groups

The results for continuous variables are shown as either means (± standard deviation) or medians (with interquartile ranges). Groups were compared using either Student’s *t* test or the Mann–Whitney *U* test, as appropriate. For categorical variables, the percentage of patients in each category was compared using a chi-square test or Fisher’s exact test. The overall time course for vital signs and FiO_2_ was compared using two-way analysis of variance for repeated measures.

#### Risk factors associated with hypoxemia during bronchoscopy

The independent predictors were assessed for risk factors associated with hypoxemia during bronchoscopy in the modified HFNC group via a univariate analysis. The statistically significant (*p* ≤ 0.1) variables from the univariate analysis were included in a multivariate analysis. The multivariate analysis was assessed using multiple logistic regression based on backward stepwise selection. We used a receiver operating characteristic (ROC) curve to confirm the cutoff value of patients with hypoxemia in the modified HFNC group.

All *p* values were two-sided, and values < 0.05 were considered significant. Data were analyzed using statistical software (SPSS 21.0; IBM Corp., Armonk, NY, USA).

## Results

### Patients

**Fig. 2:**
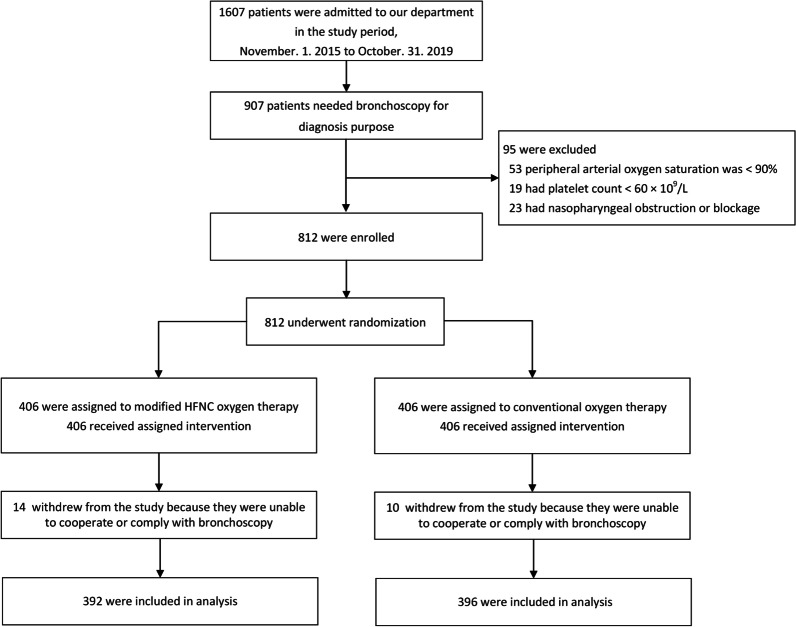
Flow diagram of the trial. *HFNC* high-flow nasal cannula

There were a total of 907 patients with indications for diagnostic bronchoscopy from November 2015 to October 2019, of whom 812 met the inclusion criteria and 95 were excluded. The remaining 812 patients were randomized to the modified HFNC (n = 406) or COT group (n = 406). Among the patients in the modified HFNC and COT groups, 14 and 10 could not cooperate and comply with bronchoscopy, respectively. Thus, 788 patients were included in the final analysis (Fig. 2).

The general clinical characteristics and physiologic parameters of the patients at the time of randomization are summarized in Table 1. The main indications for bronchoscopy were suspected lung cancer (33.0%), nonrevolving pneumonia (20.9%), and interstitial lung disease (20.1%). The arterial blood gas values at the time of randomization were normal. Baseline spirometry revealed the ratio of forced expiratory volume in one second (FEV1) to forced vital capacity (FVC), and the percent of predicted diffusing capacity of lungs for carbon monoxide (DLco) was 73.4% and 78.1% lower than normal, respectively. There were no significant differences between the two groups with respect to age, sex, body mass index (BMI), smoking history, indication for bronchoscopy, vital signs, arterial blood gas values, baseline spirometry, or laboratory parameters.

**Table 1:** Characteristics of patients at randomization

Characteristic	All patients (n = 788)	Modified high-flow nasal cannula oxygen group therapy (n = 392)	Conventional oxygen therapy group (n = 396)
Age (years)	58.5 (49.0–66.0)	58.0 (50.0–66.0)	59.0 (48.0–66.0)
Male, no. (%)	426 (54.1)	204 (52.4)	222 (56.1)
Body mass index (kg/m^2^)	23.7 (21.1–26.0)	23.7 (21.3–26.0)	23.6 (21.0–26.1)
Current smoker, no. (%)	173 (22.0)	85 (21.7)	88 (22.2)
Pack-years no	30.0 (15.0–45.0)	30.0 (13.5–50.0)	30.0 (15.0–40.0)
Indication for bronchoscopy, no. (%)			
Hemoptysis	87 (11.0)	40 (10.2)	47 (11.9)
Unexplained chronic cough	39 (4.9)	22 (5.6)	17 (4.3)
Non-resolving pneumonia	165 (20.9)	78 (19.9)	87 (22.0)
Pneumonia in immunocompromised host	71 (9.0)	41 (10.5)	30 (7.6)
Interstitial lung disease	158 (20.1)	85 (21.7)	73 (18.4)
Suspected lung cancer	260 (33.0)	123 (31.4)	137 (34.6)
Foreign body aspiration	8 (1.0)	3 (0.8)	5 (1.3)
Vital signs			
Temperature, °C	36.6 ± 0.51	36.6 ± 0.49	36.6 ± 0.53
Respiratory rate (beats/min)	17 ± 4	17 ± 5	18 ± 4
Heart rate (beats/min)	84 ± 15	84 ± 15	83 ± 15
Mean arterial pressure (mmHg)	93 ± 17	93 ± 20	94 ± 13
Arterial blood gas (room air)			
pH	7.42 (7.40–7.44)	7.42 (7.40–7.44)	7.42 (7.39–7.44)
PaO_2_ (mmHg)	79.0 (71.0–88.0)	80.0 (70.0–89.0)	79.0 (71.0–88.0)
PaCO_2_ (mmHg)	40.0 (37.0–43.0)	40.0 (37.0–43.0)	40.0 (37.0–43.0)
HCO_3_^−^ (mmol/L)	24.4 (21.8–27.2)	24.6 (22.2–26.9)	23.8 (21.4–27.9)
SaO_2_ (%)	96.0 (94.0–97.0)	96.0 (94.0–97.0)	96.0 (94.0–97.0)
Baseline spirometry			
FVC (L)	3.08 ± 0.95	3.13 ± 0.91	3.03 ± 0.98
FEV1 (L)	2.32 ± 0.78	2.36 ± 0.74	2.28 ± 0.82
FEV1 (% predicted)	82.7 ± 12.7	81.3 ± 13.3	83.9 ± 12.2
FEV1/FVC (%)	73.4 ± 12.8	73.7 ± 12.0	73.2 ± 13.2
DLco (% predicted)	78.1 ± 9.9	77.9 ± 9.5	78.4 ± 10.3
Laboratory parameters			
White blood cell (× 10^9^/L)	6.59 (5.23–8.27)	6.34 (5.07–8.25)	6.79 (5.34–8.33)
Platelet count (× 10^9^/L)	237 (194–293)	232 (192–288)	242 (196–300)
Prothrombin time (s)	11.2 (9.9–12.6)	11.1 (10.0–12.4)	11.3 (9.8–12.7)
Fibrinogen (mg/dL)	285.1 ± 104.1	283.6 ± 104.6	286.6 ± 103.7
C-reactive protein (mg/dL)	0.8 (0.5–1.2)	0.8 (0.5–1.1)	0.8 (0.5–1.2)
Procalcitonin (pg/ml)	0.10 (0.08–0.12)	0.10 (0.08–0.11)	0.10 (0.08–0.12)

PaO_2_, partial pressure of arterial oxygen; PaCO_2_, partial pressure of arterial carbon dioxide; HCO_3_^−^, bicarbonate; SaO_2_, arterial oxygen saturation; FVC, forced vital capacity; FEV1, forced expiratory volume in one second; DLco, diffusing capacity of lungs for carbon monoxide

### Endpoints

Clinical endpoints of the patients are shown in Table 2. The proportion of patients with a single moment of SpO_2_ < 90% during bronchoscopy in the modified HFNC group was significantly lower than that in the COT group (12.5% vs. 28.8%,* p* < 0.001). The duration of bronchoscopy was 685 s (range 485–850 s) in the modified HFNC group, which was significantly shorter than the 800 s (range 614–990 s) in the COT group (*p* < 0.001). The duration of SpO_2_ < 90% was significantly shorter in the HFNC group. Furthermore, 102 (25.8) patients had procedural discontinuation in the COT group, while only 39 (9.9) patients in the HFNC group (*p* < 0.001).

**Table 2:** Clinical endpoints, bronchoscopy-related events, and complications according to study group

Outcome	All patients (n = 788)	Modified high-flow nasal cannula oxygen group therapy (n = 392)	Conventional nasal cannula oxygen group (n = 396)	*P*
Primary endpoint				
The number of patients with a single moment of SpO_2_ < 90% during bronchoscopy, no. (%)	163 (20.7)	49 (12.5)	114 (28.8)	< 0.001
Secondary endpoint				
Duration of bronchoscopy (s)	780 (592–965)	685 (485–850)	800 (614–990)	< 0.001
Other endpoints				
Duration of SpO_2_ < 90% (s)	98 (29–130)	22 (14–27)	115 (96–137)	< 0.001
The number of patients with procedural discontinuation no. (%)	141 (17.9)	39 (9.9)	102 (25.8)	< 0.001
Bronchoalveolar lavage				
Volume of fluid instilled (ml)	60 (40–100)	60 (40–100)	60 (40–100)	0.751
Volume of fluid recovered (ml)	20 (15–45)	22 (15–45)	20 (15–45)	0.483
Bronchial brushing, no. (%)	736 (93.4)	367 (93.6)	369 (93.2)	0.803
Endobronchial biopsy, no. (%)	677 (85.9)	337 (86.0)	340 (85.9)	0.964
Transbronchial lung biopsy, no. (%)	235 (29.8)	115 (29.3)	120 (30.3)	0.767
Events during bronchoscopy, no. (%)				
Agitation	119 (15.1)	43 (11.0)	76 (19.2)	0.001
Bronchospasm	30 (3.8)	12 (3.1)	18 (4.5)	0.276
Arrhythmias				0.559
Ventricular arrhythmias	8 (1.0)	3 (0.8)	5 (1.3)	0.484
Supraventricular tachycardias	16 (2.0)	4 (1.0)	12 (3.0)	0.045
Premature atrial contractions	25 (3.2)	11 (2.8)	14 (3.5)	0.559
Premature ventricular contraction	14 (1.8)	5 (1.3)	9 (2.3)	0.289
Hypertension (systolic blood pressure > 180 mmHg)	124 (15.7)	64 (16.3)	60 (15.2)	0.651
Epistaxis	40 (5.1)	18 (4.6)	22 (5.6)	0.538
Mucosal bleeding	204 (25.9)	103 (26.3)	101 (25.5)	0.805
Complications of bronchoscopy, no. (%)				
Fever	162 (20.6)	78 (19.9)	84 (21.2)	0.648
Pneumothorax	33 (4.2)	10 (2.6)	23 (5.8)	0.022
Hemorrhage	51 (6.5)	25 (6.4)	26 (6.6)	0.915

SpO_2_, peripheral arterial oxygen saturation

There were no significant differences in the volume of instilled and retrieved fluid, number of bronchial brushings, endobronchial biopsies, or transbronchial lung biopsies between the two groups. Significantly fewer patients had agitation (11.0% vs. 19.2%,* p* = 0.001) and supraventricular tachycardias (1.0% vs. 3.0%,* p* = 0.045) in the modified HFNC group than in the COT group. Pneumothorax occurred in the COT group with a higher prevalence than in the modified HFNC group (5.8% vs. 2.6%,* p* = 0.022). There were no significant differences in other complications between the two groups.

### Time course of vital signs and FiO_2_

**Fig. 3:**
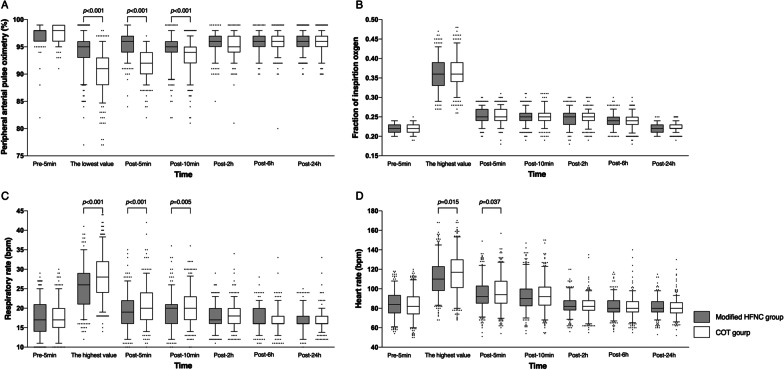
Comparison of vital signs and FiO_2_ between the modified HFNC and COT groups. *HFNC* high-flow nasal cannula, *COT* conventional oxygen therapy

Vital signs and FiO_2_ were monitored before, during, and after bronchoscopy. There were no significant differences in FiO_2_ between the two groups. The SpO_2_ was significantly decreased, and the FiO_2_, respiratory rate, heart rate, and mean arterial pressure were significantly increased during bronchoscopy in both groups (Table 3 and Fig. 3). During and 5 min after bronchoscopy, the SpO_2_ in the modified HFNC group was significantly higher than that in the COT group, and the respiratory and heart rates were significantly lower than those in the COT group. Two hours after bronchoscopy, the respiratory and heart rates and the mean arterial pressure returned to the levels before bronchoscopy, and the SpO_2_ slowly recovered.

**Table 3:** Comparison of vital signs between the modified high-flow nasal cannula oxygen and the conventional oxygen therapy groups

Characteristic	Group	Pre-5 min (n = 392/396)	The lowest SpO_2_, the highest respiratory rate, heart rate, and mean arterial pressure (n = 392/396)	Post-5 min (n = 392/396)	Post-10 min (n = 392/396)	Post-2 h (n = 392/396)	Post-6 h (n = 392/396)	Post-24 h (n = 392/396)	*p*^*a*^
SpO_2_ (%)	MHFNC	97.4 ± 1.6	94.1 ± 3.2	95.4 ± 2.3	94.7 ± 2.7	95.5 ± 1.8	95.7 ± 1.8	96.0 ± 1.7	< 0.001
	Control	97.3 ± 1.5	90.5 ± 3.8	92.1 ± 2.6	93.4 ± 2.9	95.3 ± 2.1	95.7 ± 1.9	95.9 ± 1.8	< 0.001
	*p*^*c*^	0.268	< 0.001	< 0.001	< 0.001	0.348	0.869	0.493	0.003^*b*^
Respiratory rate (bpm)	MHFNC	17 ± 5	26 ± 7	19 ± 5	19 ± 4	18 ± 3	17 ± 3	17 ± 3	< 0.001
	Control	18 ± 4	28 ± 6	21 ± 5	20 ± 4	18 ± 3	17 ± 3	17 ± 3	< 0.001
	*p*^*c*^	0.196	< 0.001	< 0.001	0.005	0.693	0.694	0.973	0.001^*b*^
Heart rate (beats/min)	MHFNC	84 ± 15	112 ± 18	94 ± 16	93 ± 16	83 ± 10	82 ± 10	81 ± 10	< 0.001
	Control	83 ± 15	116 ± 21	97 ± 18	94 ± 16	82 ± 10	81 ± 11	80 ± 10	< 0.001
	*p*^*c*^	0.305	0.015	0.037	0.377	0.167	0.818	0.334	0.465^*b*^
Mean arterial pressure (mmHg)	MHFNC	95 ± 13	108 ± 15	102 ± 14	104 ± 17	95 ± 10	94 ± 10	94 ± 9	< 0.001
	Control	94 ± 12	110 ± 18	103 ± 16	104 ± 16	96 ± 10	95 ± 10	94 ± 10	< 0.001
	*p*^*c*^	0.454	0.256	0.233	0.890	0.541	0.342	0.580	0.402^*b*^

FiO_2_ the fraction of inspired oxygen, SpO_2_ peripheral arterial oxygen saturation

*p*^*a*^ for overall comparisons of differences in each group over time

*p*^*b*^ for overall comparisons of differences between groups over time

*p*^*c*^ for comparisons of differences between groups at each time point

### Risk factors associated with hypoxemia in the modified HFNC group

Forty-nine of 392 patients in the modified HFNC group were hypoxemic during bronchoscopy. Based on multivariate logistic regression analysis, a baseline FVC < 2.7 L (OR, 0.276; 95% CI, 0.083–0.919, *p* = 0.036) and volume of fluid instilled > 60 ml (OR, 1.034; 95% CI, 1.002–1.067, *p* = 0.036) were identified as independent risk factors associated with hypoxemia during bronchoscopy in the modified HFNC group (Table 4).

**Table 4:** Risk factors associated with hypoxemia during bronchoscopy in modified high-flow nasal cannula oxygen group

Variable	β coefficient	Standard error	Odds ratios (95% *CI*)	*P*
Univariate logistic regression				
Baseline PaO_2_	− 0.027	0.012	0.973 (0.950–0.996)	0.024
Baseline SaO_2_	− 0.199	0.069	0.820 (0.716–0.939)	0.004
Baseline FVC	− 1.027	0.456	0.356 (0.147–0.875)	0.024
Volume of fluid instilled	0.011	0.004	1.012 (1.003–1.020)	0.005
Hemorrhage	0.715	0.337	2.044 (1.055–3.959)	0.034
Multivariate logistic regression				
Baseline FVC	− 1.286	0.613	0.276 (0.083–0.919)	0.036
Volume of fluid instilled	0.034	0.016	1.034 (1.002–1.067)	0.036

PaO_2_, partial pressure of arterial oxygen; SaO_2_, arterial oxygen saturation; FVC, forced vital capacity

## Discussion

In this study, we showed that, compared with oxygen therapy by nasal cannula, modified HFNC significantly prevented the incidence of hypoxia and shortened the duration of bronchoscopy. The modified HFNC reduced the occurrence of agitation and arrhythmias during bronchoscopy. In addition, baseline FVC < 2.7 L and volume of fluid instilled > 60 ml were independently associated with hypoxemia during bronchoscopy.

HFNC oxygen therapy has become increasingly popular in the treatment of patients with various clinical conditions [17]. It has been proposed that HFNC oxygen therapy provides several benefits [18]. Among these benefits are maintenance of a constant FiO_2_, generation of a positive end-expiratory pressure (PEEP), reduction in the anatomic dead space, improvement of mucociliary clearance, and decreased work of breathing.

As a new type of oxygen therapy, several studies have involved the use of HFNC as a support for oxygen supplementation during bronchoscopy. A recent study showed that HFNC provides better oxygenation than standard therapy, prevents lung de-recruitment, and avoids the increase of diaphragm activation during bronchoscopy for BAL. Compared with the standard therapy group, fewer desaturations occurred (11% vs. 56%; *p* < 0.01) in the HFNC group [19]. It aligns with our observation. In patients undergoing endobronchial ultrasound-guided transbronchial needle aspiration (EBUS-TBNA), HFNC could prevent desaturation compared with standard oxygen therapy [20, 21]. In addition, HFNC can also be used for lung transplant patients to reduce the proportion of hypoxemia during bronchoscopy [22].

HFNC oxygen therapy, however, has some shortcomings with respect to transnasal bronchoscopy. HFNC oxygen therapy is applied to both nostrils, and the narrowing of the lumen influences the flow in the nostril in which the bronchoscope is inserted. Lower flow affects the level of PEEP generated [23]. Moreover, when the patient’s inspiratory flow rates exceed the flow delivered during bronchoscopy, the additional flow is recruited from the surrounding air (FiO_2_ = 0.21). In this situation, the inspired FiO_2_ is significantly lower than that of the delivered gas [24]. Therefore, we designed a modified HFNC that has a single cannula. The bronchoscope passes through one nostril of the patient, and modified HFNC oxygen therapy is given through the contralateral nostril. Compared with the unmodified version of the HFNC, the air flow passes through the single nasal cannula faster with the same flow rate. Based on the results of an in vitro study (Additional file 1), the modified HFNC has similar respiratory support characteristics as the regular HFNC under different respiratory conditions. Furthermore, the modified HFNC provides higher PEEP at high-flow rates.

Hypoxemia during bronchoscopy is common [25]. Due to partial occlusion of the airway by the bronchoscope, respiratory mechanics are altered [26]. As a result, oxygen saturation may decrease to < 90% despite oxygen supplementation. In the current study, compared with the nasal cannula, the modified HFNC decreased the incidence of hypoxemia during bronchoscopy. It is conceivable that two factors accounted for this finding. First, the FiO_2_ value was more stable and reliable because of reduced losses and the minimization of ambient air entrainment [27]. Second, the PEEP generated by high-flow rates may prevent alveolar collapse, improving dynamic compliance and oxygenation. This study is of certain clinical relevance, but the modified HFNC cannot entirely resolve the problem of hypoxemia during bronchoscopy. Although the FiO_2_ in the COT group was estimated rather than accurately measured, the results of the empirical formula are relatively accurate at low flow rates. We have also noticed that FiO_2_ is highly variable with breathing changes that result in greater amounts of entrained room air (increased respiratory rate, tidal volume, and inspiratory force) in the COT group. In theory, COT can only provide 24%-45% oxygen with flow rates up to 6 L/min. In order that the COT was comparable to modified HFNC, we did not adjust the FiO_2_ very high in the modified HFNC group during bronchoscopy (e.g., > 0.5). Using approximately the same FiO_2_, the modified HFNC was able to stabilize oxygenation maintenance during bronchoscopy, avoiding discontinuation of the procedure due to hypoxemia. Therefore, the duration of bronchoscopy was shorter in the modified HFNC group. Moreover, fewer patients with agitation could decrease the frequency of pneumothorax.

NIV can also prevent hypoxemia during bronchoscopy, but facemask intolerance and difficulty manipulating the scope through the mask limit its appeal. Previous studies have shown that NIV provides greater adequacy and stability of oxygenation than HFNC treatment in hypoxemic patients undergoing bronchoscopy [10, 13]. Therefore, NIV treatment provides better effectiveness for oxygen supplementation during bronchoscopy in patients with hypoxemia. Nevertheless, HFNC therapy is more comfortable and easier for bronchoscopists to apply than NIV.

Fiberoptic bronchoscopy with BAL is an important tool for determining the etiology of pneumonia, but BAL has significant risks for oxygenation deterioration [28]. In the current study, a > 60 ml volume of fluid instilled was identified as an independent risk factor associated with hypoxemia during bronchoscopy in the modified HFNC group. A large volume of BAL is bound to affect gas exchange, which leads to hypoxemia in patients. Various studies, mainly involving hypoxemic patients, also showed that BAL may worsen the decrease in PaO_2_ during bronchoscopy [29, 30]. In addition, we found that a baseline FVC < 2.7 L was an independent risk factor for hypoxemia during bronchoscopy in the modified HFNC group. Patients with a lower FVC generally had different chronic respiratory diseases, and these patients were prone to hypoxemia during bronchoscopy. Therefore, we believe that an adequate baseline FVC is necessary to avoid hypoxemia during bronchoscopy.

Mildly hypoxemic participants included in our study had SpO_2_ > 90%, and the FiO_2_ was adjusted in a timely manner during bronchoscopy. In addition, all bronchoscopy procedures were performed by three experienced respiratory specialists. Therefore, the incidence of hypoxemia in all patients was only 20.7%.

Guidelines suggest sedation should be offered to patients [6]. However, all patients in our study were given topical anesthesia, but not intravenous sedation. Our approach is similar to previous studies using topical anesthesia instead of sedation [19, 31]. The sedative can affect the airway tone and respiratory drive. Avoiding the use of sedatives in our study, we ruled out the confounding factors that could influence gas exchange.

Our trial had several limitations. First, the study had a single-blind design. Therefore, chance or unintentional treatment decision bias could not be completely eliminated; however, the treatments were strictly implemented according to the protocols in each group. Second, our study did not include patients with acute respiratory failure; thus, the benefit of the modified HFNC for such patients could not be determined and will be included in future work. Third, there were more patients with agitation and pneumothorax in the COT group. Increased agitation and pneumothorax may also have contributed to more hypoxemic events. Therefore, these confounders affect the duration of bronchoscopy. Forth, the FiO_2_ estimated by the empirical formula in the COT group may be inaccurate, particularly in the setting of increased work of breathing and introduction of the bronchoscope. Fifth, this study lacks exploring the physiological mechanism of avoiding hypoxemia by HFNC, such as the change of end-expiratory lung volume during bronchoscopy.

## Conclusions

In conclusion, the findings from this randomized controlled trial suggest that a modified HFNC could decrease the proportion of patients with a single moment of SpO_2_ < 90% during bronchoscopy and shorten the duration of bronchoscopy. A lower baseline FVC and large-volume BAL may predict hypoxemia during bronchoscopy with a modified HFNC. However, due to study limitations, high-quality randomized controlled trials further investigating the efficacy and safety of modified HFNC therapy in acute respiratory failure patients are warranted.

## Supplementary Information

**Additional file 1.** 1. Methods. 1.1 Appendix S1: Assessment of the modified HFNC in vitro test. 2. Tables. 2.1 Table S1: The combination of parameters defines a state by TestChest. 2.2 Table S2: Effect of modified and regular HFNC on PEEP. 2.3 Table S3: Effect of modified and regular HFNCs on tidal volumes. 2.4 Table S4: Effect of modified and regular HFNCs on FiO2 (FiO2 set at 50%). 3.1 Figure S1: Modified high-flow nasal cannula. 3.2 Figure S2: Device connection diagram.

## Data Availability

The datasets used and/or analyzed during the current study are available from the corresponding author on reasonable request.

## Abbreviations

PaO_2_: Partial pressure of arterial oxygen
BAL: Bronchoalveolar lavage
SaO_2_: Arterial oxygen saturation
NIV: Noninvasive ventilation
HFNC: High-flow nasal cannula
SpO_2_: Peripheral arterial oxygen saturation
COT: Conventional oxygen therapy
FiO_2_: Fraction of inspired oxygen
BMI: Body mass index
ROC: Receiver operating characteristic
FEV1: Forced expiratory volume in one second
FVC: Forced vital capacity
DLco: Diffusing capacity of lungs for carbon monoxide
PEEP: Positive end-expiratory pressure
EBUS-TBNA: Endobronchial ultrasound-guided transbronchial needle aspiration
