# Supplementary material for: Modified high-flow nasal cannula oxygen therapy versus conventional oxygen therapy in patients undergoing bronchoscopy: a randomized clinical trial
Source: BMC Pulm Med. 2021 Nov 14;21:367. doi: 10.1186/s12890-021-01744-8 (PMC8591908; doi:10.1186/s12890-021-01744-8)
Supplement: Supplementary file 1 — Additional file 1. 1. Methods. 1.1 Appendix S1: Assessment of the modified HFNC in vitro test. 2. Tables. 2.1 Table S1: The combination of parameters defines a state by TestChest. 2.2 Table S2: Effect of modified and regular HFNC on PEEP. 2.3 Table S3: Effect of modified and regular HFNCs on tidal volumes. 2.4 Table S4: Effect of modified and regular HFNCs on FiO2 (FiO2 set at 50%). 3.1 Figure S1: Modified high-flow nasal cannula. 3.2 Figure S2: Device connection diagram. [file 12890_2021_1744_MOESM1_ESM.docx]

**Supplemental Digital Content:**

**1. Supplemental Methods**

1.1 Appendix S1: Assessment of the modified HFNC *in vitro* test

**2. Supplemental Tables**

2.1 Table S1: The combination of parameters defines a state by TestChest

2.2 Table S2: Effect of modified and regular HFNC on PEEP

2.3 Table S3: Effect of modified and regular HFNCs on tidal volumes

2.4 Table S4: Effect of modified and regular HFNCs on FiO_2_ (FiO_2_ set at 50%)

**3. Supplemental Figures**

3.1 Figure S1: Modified high-flow nasal cannula

3.2 Figure S2: Device connection diagram

**1.1 Appendix S1: Assessment of the modified HFNC *in vitro* test**

In our center the bronchoscope is passed through the nose in all procedures. Bronchoscopy will occupy one of the nares receiving oxygen therapy during the bronchoscopy. For this reason, we designed a modified high-flow oxygen therapy (HFNC) that has a single cannula (Figure S1). The modified high-flow nasal cannula has been awarded a patent in China (ZL 2015 2 0748440.5). Before being used in clinical trials, the modified HFNC was assessed by an in vitro test.

**Methods**

**Device connection**

As shown in eFigure 1, AIRVO^TM^ 2 (Fisher & Paykel Healthcare, Auckland, New Zealand) was equipped with the modified and a regular nasal cannula (OPT844 nasal cannula; Fisher & Paykel Healthcare). The medium size of the OPT844 nasal cannula matched the size of the manikin. An airway model (Airsim Advance Combo; Trucorp, Craigavon, North Ireland) was connected to a test lung (TestChest; Organis GmbH, Landquart, Switzerland). AIRVO 2, with the different nasal interfaces, was used to apply high-flow oxygen therapy for the model. Different parameters were set to define a state by TestChest through the computer, then collected data.

**Device setting**

For the AIRVO 2 system, the humidifier temperature was set at 37°C, the fraction of inspired oxygen (FiO_2_) was set at 50%, and the flow rates were 30, 40, and 50 L/min. The combination of parameters was entered in the TestChest control software (TestChest Basic Control software, version 1.81). Occlusion pressure at 100 ms (P0.1), airways resistance (R_aw_), respiratory system compliance (C_rs_), chest wall compliance (C_cw_), and functional residual capacity (FRC) were set for the TestChest to simulate normal breathing, rapid shallow breathing, and deep breathing, as well as acute respiratory distress syndrome (ARDS) and chronic obstructive pulmonary disease (COPD) states (eTable 1).

**Measurement**

The positive end expiratory pressure (PEEP), tidal volumes, and FiO_2_ were measured during closed mouth status. For each setting, data were collected 10 times after stabilization for 10 min.

**Results**

Under different respiratory conditions, the modified HFNC PEEP was significantly higher than the regular HFNC at a flow rate of 50 L/min (eTable 2). There was no significant difference in PEEP between the two types of nasal cannula at other flow rates. There was no significant difference in tidal volume and FiO_2_ between the modified and regular HFNC under different simulated conditions and flow rates (eTable 3 and eTable 4).

**Conclusions**

In conclusion, the modified HFNC has similar respiratory support characteristics as the regular HFNC, but can provide higher PEEP at high-flow rates.

**2. Supplemental Tables**

2.1 Table S1: The combination of parameters defines a state by TestChest

|  | P0.1 | R_aw_ | C_rs_ | C_cw_ | FRC |
| --- | --- | --- | --- | --- | --- |
| Normal breathing | 5 | 5 | 60 | 120 | 2100 |
| Rapid shallow breathing | 3 | 5 | 60 | 120 | 2100 |
| Deep breathing | 8 | 5 | 60 | 120 | 2100 |
| ARDS | 8 | 5 | 30 | 80 | 1000 |
| COPD | 8 | 50 | 30 | 80 | 2500 |

P0.1 occlusion pressure at 100 ms, R_aw_ airways resistance, C_rs_ respiratory system compliance, C_cw_ chest wall compliance, FRC functional residual capacity, ARDS acute respiratory distress syndrome, COPD chronic obstructive pulmonary disease

2.2 Table S2: Effect of modified and regular HFNC on PEEP

|  | Flow (L/min) | Modified HFNC | Regular HFNC | p |
| --- | --- | --- | --- | --- |
| Normal breathing |  |  |  |  |
|  | 30 | 0.67±0.09 | 0.70±0.02 | 0.199 |
|  | 40 | 1.00±0.10 | 1.06±0.15 | 0.207 |
|  | 50 | 1.85±0.07 | 1.62±0.07 | <0.001 |
| Rapid shallow breathing |  |  |  |  |
|  | 30 | 0.64±0.10 | 0.57±.012 | 0.098 |
|  | 40 | 1.10±0.10 | 1.03±0.12 | 0.106 |
|  | 50 | 1.76±0.12 | 1.57±0.18 | 0.007 |
| Deep breathing |  |  |  |  |
|  | 30 | 0.61±0.18 | 0.64±0.10 | 0.579 |
|  | 40 | 1.15±0.03 | 1.13±0.05 | 0.274 |
|  | 50 | 1.85±0.06 | 1.64±0.10 | <0.001 |
| ARDS |  |  |  |  |
|  | 30 | 0.57±0.15 | 0.59±0.12 | 0.747 |
|  | 40 | 1.16±0.10 | 1.12±0.06 | 0.209 |
|  | 50 | 1.92±0.03 | 1.83±0.13 | 0.025 |
| COPD |  |  |  |  |
|  | 30 | 0.72±0.12 | 0.75±0.14 | 0.531 |
|  | 40 | 1.21±0.20 | 1.14±0.05 | 0.263 |
|  | 50 | 2.10±0.09 | 1.65±0.27 | <0.001 |

HFNC high-flow nasal cannula, PEEP positive end expiratory pressure, ARDS acute respiratory distress syndrome, COPD chronic obstructive pulmonary disease

2.3 Table S3: Effect of modified and regular HFNCs on tidal volumes

|  | Flow (L/min) | Modified HFNC | Regular HFNC | p |
| --- | --- | --- | --- | --- |
| Normal breathing |  |  |  |  |
|  | 30 | 326±1 | 326±3 | 0.386 |
|  | 40 | 339±3 | 338±4 | 0.488 |
|  | 50 | 386±3 | 385±3 | 0.130 |
| Rapid shallow breathing |  |  |  |  |
|  | 30 | 264±3 | 264±2 | 0.783 |
|  | 40 | 287±1 | 288±1 | 0.201 |
|  | 50 | 320±2 | 322±4 | 0.268 |
| Deep breathing |  |  |  |  |
|  | 30 | 353±3 | 354±3 | 0.271 |
|  | 40 | 374±4 | 373±4 | 0.709 |
|  | 50 | 411±2 | 412±2 | 0.430 |
| ARDS |  |  |  |  |
|  | 30 | 114±2 | 114±1 | 0.738 |
|  | 40 | 128±1 | 128±1 | 0.835 |
|  | 50 | 153±1 | 153±1 | 0.811 |
| COPD |  |  |  |  |
|  | 30 | 385±1 | 387±1 | 0.137 |
|  | 40 | 414±3 | 414±3 | 0.711 |
|  | 50 | 454±2 | 455±2 | 0.123 |

HFNC high-flow nasal cannula, ARDS acute respiratory distress syndrome, COPD chronic obstructive pulmonary disease

2.4 Table S4: Effect of modified and regular HFNCs on FiO_2_ (FiO_2_ set at 50%)

|  | Flow (L/min) | Modified HFNC | Regular HFNC | p |
| --- | --- | --- | --- | --- |
| Normal breathing |  |  |  |  |
|  | 30 | 50.6±0.5 | 50.5±0.7 | 0.780 |
|  | 40 | 50.6±0.4 | 50.6±0.8 | 0.926 |
|  | 50 | 51.8±0.5 | 51.9±0.5 | 0.627 |
| Rapid shallow breathing |  |  |  |  |
|  | 30 | 50.0±0.9 | 50.5±1.1 | 0.251 |
|  | 40 | 49.5±0.4 | 49.6±0.4 | 0.708 |
|  | 50 | 49.8±0.6 | 49.7±0.5 | 0.972 |
| Deep breathing |  |  |  |  |
|  | 30 | 49.8±0.7 | 49.6±0.5 | 0.408 |
|  | 40 | 50.1±0.9 | 49.5±1.0 | 0.153 |
|  | 50 | 51.1±0.7 | 51.1±0.3 | 0.879 |
| ARDS |  |  |  |  |
|  | 30 | 50.4±0.7 | 50.3±0.7 | 0.637 |
|  | 40 | 49.8±0.4 | 49.7±0.4 | 0.663 |
|  | 50 | 49.9±0.2 | 50.0±0.3 | 0.365 |
| COPD |  |  |  |  |
|  | 30 | 50.4±0.4 | 50.3±0.3 | 0.405 |
|  | 40 | 51.3±0.4 | 51.1±0.3 | 0.290 |
|  | 50 | 52.5±0.5 | 52.5±0.4 | 0.790 |

HFNC high-flow nasal cannula, FiO_2_ fraction of inspired oxygen, ARDS acute respiratory distress syndrome, COPD chronic obstructive pulmonary disease

**3. Supplemental Figures**

3.1 Figure S1: Modified high-flow nasal cannula


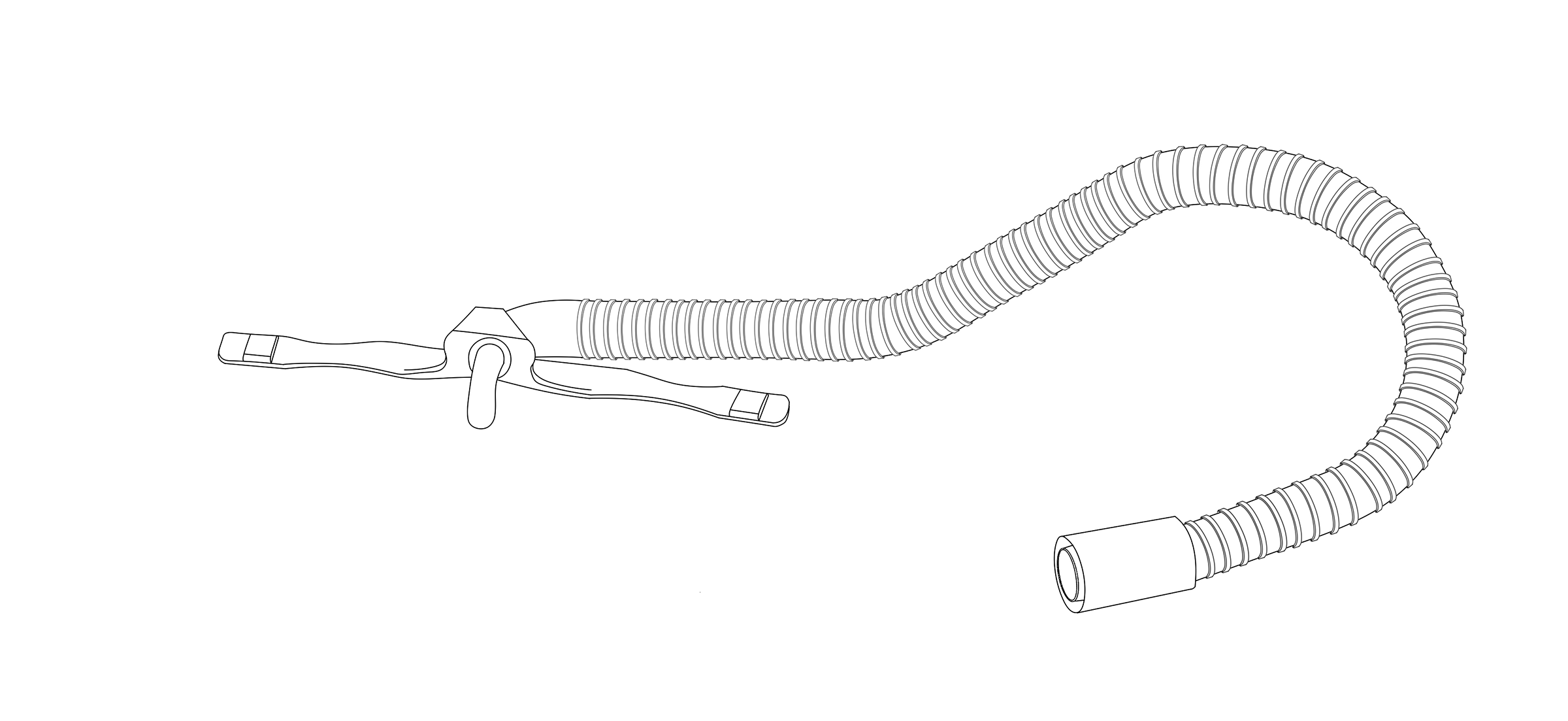


3.2 Figure S2: Device connection diagram


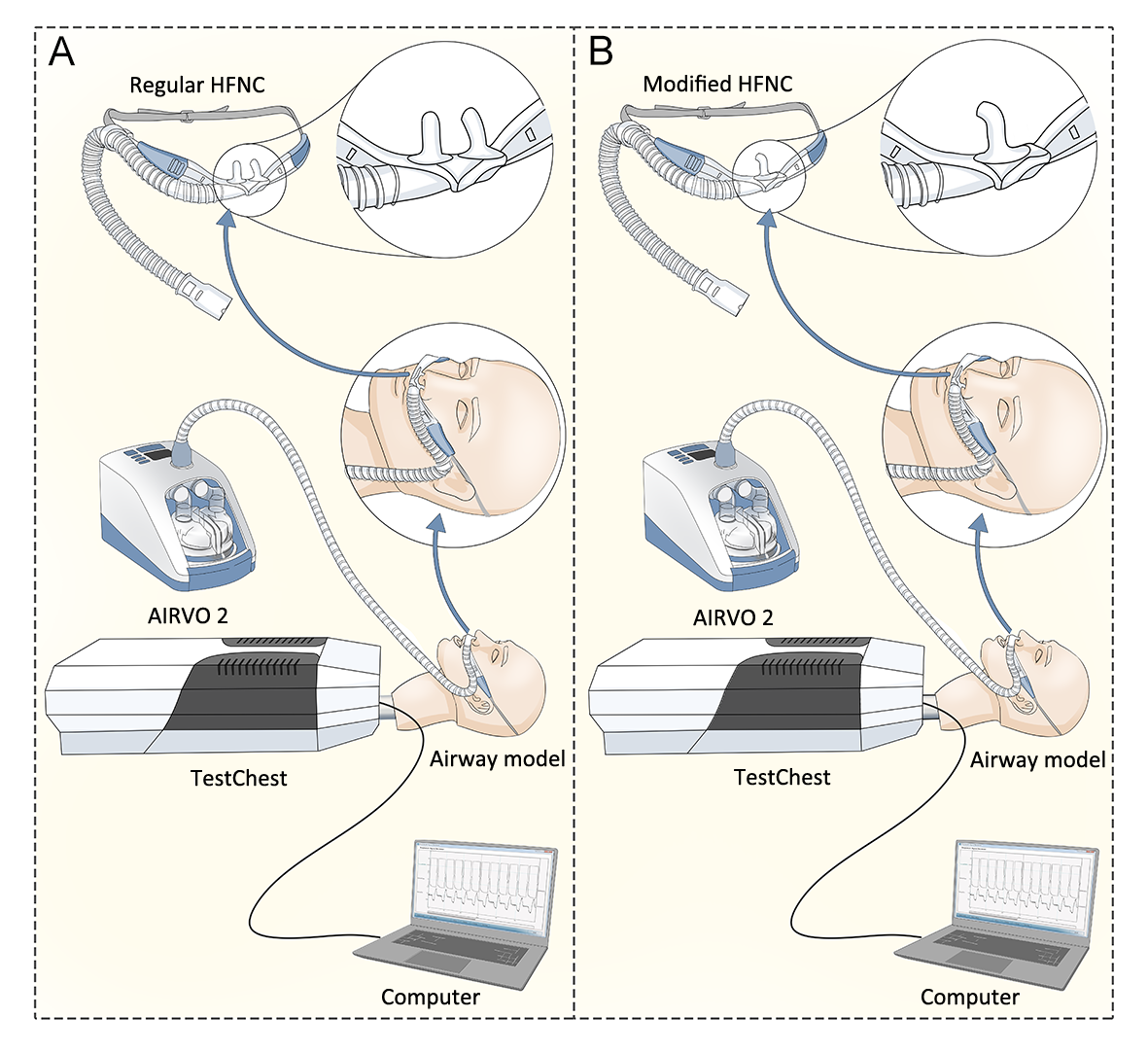


A Regular HFNC. B Modified HFNC

HFNC high-flow nasal cannula

AIRVO 2 was equipped with the modified and a regular nasal cannula. An airway model was connected to TestChest. AIRVO 2, with the different nasal interfaces, was used to apply high-flow oxygen therapy for the model. Different parameters were set to define a state by TestChest through the computer, then collected data.
